# Protein and Polysaccharide Complexes for Alleviating Freeze-Induced Damage in Sour Cream and Yogurt

**DOI:** 10.3390/foods14244193

**Published:** 2025-12-06

**Authors:** Ripley Vaughan, Vermont Dia, Elizabeth Eckelkamp, Tong Wang

**Affiliations:** 1 Department of Food Science, University of Tennessee, 2510 River Drive, Knoxville, TN 37996, USA; 2 Department of Animal Science, University of Tennessee, 2510 River Drive, Knoxville, TN 37996, USA

**Keywords:** freeze-induced damage, protein–polysaccharide complexes, sour cream, yogurt, ice recrystallization inhibition

## Abstract

There has been little research on freezing-induced damage in high-moisture dairy products, specifically sour cream and yogurt. This work aimed to investigate, as a proof-of-concept, if antifreeze additives may prevent quality decrease in high-moisture dairy products due to freeze-induced damage. Whey protein isolate and soy protein isolate were complexed with locust bean gum and lambda carrageenan, in both unhydrolyzed and hydrolyzed forms, and their antifreeze activity was evaluated in a model system as well as in sour cream and yogurt. The biomolecules were also tested individually as controls to determine any synergistic effects. Protein and polysaccharide complexes were found to have ice recrystallization inhibition activity in the model systems by reducing the ice crystal size significantly (35–64%) compared to the negative control at both pH 4.5 and 7.0. However, the complexes failed to prevent freeze-induced damage in the dairy system and all treatments resulted in decreased firmness, cohesiveness, and consistency along with increased graininess, possibly due to the complex interacting with different food components that may have interfered with the antifreeze activity of the tested compounds.

## 1. Introduction

Freeze-induced structural damage is a common problem in frozen foods and results in decreased quality and increased food waste. This damage is caused by ice recrystallization and growth, volume expansion, cryo-concentration, and protein dehydration [1,2,3]. In high-moisture dairy products, the protein matrix helps structure the food system and contributes to its texture but can physically break due to ice expansion during ice recrystallization [1,4]. Proteins can become dehydrated during freezing which can reduce the protein’s ability to rebind water [4]. As bound water is released from the protein matrix during freezing there is an increase in unbound water resulting in syneresis [5]. Ice recrystallization can also cause rupture of the milk fat globule causing fat leakage from the globule [5]. Most research investigating freeze-induced damage in dairy products has been conducted using ice cream [6,7] and high- and low-moisture mozzarella cheese [2,4,8]. Although yogurt and sour cream are not commonly frozen, identifying an additive to help prevent freeze-induced damage would be beneficial in the event of supply chain disruption or the need to prolong their shelf life. In addition, these products may serve as a sensitive model to identify antifreeze agents.

Many molecules have been investigated for ice recrystallization inhibition (IRI) properties. There is a category of antifreeze proteins (AFP) naturally present in polar fish and plants that are responsible for their survival at low temperatures [9]. These proteins have potent IRI activity, but they are in low abundance and expensive to obtain [10]. This led to the need to identify molecules that are more readily available with moderate IRI activity. Synthetic polymers such as polyvinyl alcohol [11], protein and polysaccharide hydrolysates [12,13], and stabilizers have been reported to have IRI activity [14].

Proteins and polysaccharides, such as whey protein isolate (WPI), locust bean gum (LBG), carrageenans, and guar gum, are commonly added to dairy products individually to help improve textural properties such as thickening and prevention of syneresis [15,16]. In yogurt specifically, WPI, whole milk powder, and casein powders are commonly added to increase the total solids content and help create a thicker product [17]. Proteins and polysaccharides have been shown to form functional complexes through electrostatic interaction [18]. Functional properties of these biomolecules can be improved when complexed together.

Not only do these complexes have potential to retain textural properties during freezing in the dairy systems, but they also have potential to be IRI-active molecules. A study performed by Monalisa et al. [19] showed an increase in IRI activity when AFP and corn starch were combined and they hypothesized this was due to the starch’s increased interaction with the water molecules allowing the AFP to better interact with the ice surface, preventing ice crystal growth. It was also reported that an increased amphiphilicity of the biopolymers when complexed together could allow the polysaccharide to interact more with the water and the protein to better interact with the ice crystal surface therefore preventing ice recrystallization [20]. The improved hydration properties from the polysaccharides could also help prevent protein dehydration. There has also been a study that showed a synergistic effect between wheat flour and AFP on ice recrystallization [21]. Wheat flour is a complex substance containing starch, protein, other polysaccharides, and lipids. They hypothesized that the complex makeup of wheat flour along with AFP prevented ice recrystallization by synergistically interacting with the quasi-liquid layer [21].

Thus, we hypothesize for this study that the protein and polysaccharide complexes will help protect against freeze-induced damage due to their improved amphiphilicity and thus higher IRI activity. Hydrolysates of proteins with lower molecular weights have also been shown to have improved IRI activity compared to larger biomolecules [12,13,22,23]. Therefore, we also hypothesize that the complexes of hydrolyzed proteins and polysaccharides will have a greater effect than the complexes of unhydrolyzed proteins and polysaccharides due to an increased mobility in a complex food matrix to act at the ice–water interface, providing better protection against freeze-induced damage. The objective of this study was to investigate the effect of two proteins (WPI and soy protein isolate (SPI)) and two polysaccharides (LBG and lambda carrageenan (LC), one is relatively neutral and the other negatively charged) and their hydrolysate complexes on freeze-induced damage in sour cream and yogurt.

To test these hypotheses, WPI and SPI were complexed with LBG and LC through pH cycling, and the same complexing procedure was applied to their hydrolysates. A SPI and lecithin complex was also evaluated due to its enhanced IRI activity as reported by Fomich et al. [24]. The complexes were first evaluated for IRI activity by the Splat Assay in a model system before being incorporated into sour cream and yogurt since they can be feasibly made in the laboratory and are of high-water content, thus a high sensitivity for freezing-induced damages. After freezing and thawing, the damage was evaluated through texture analysis and microscopic image analysis. Lastly, further characterization of the complexes was performed using particle size analysis and qualitative emulsion stability testing.

## 2. Materials and Methods

### 2.1. Materials for Complex Preparation and Sour Cream and Yogurt Production

The proteins used for this project were WPI (90% protein) from Bulk Supplements (Las Vegas, NV, USA) and SPI ProFam 931 from ADM (Decatur, IL, USA). The polysaccharides were LBG and LC from Modernist’s Pantry (Eliot, ME, USA). The enzyme used for protein hydrolysis was Alcalase from *Bacillus licheniformis* (3.03 Au/mL) from EMD Millipore Corp. (Billerica, MA, USA). The cellulase for hydrolysis of the polysaccharides was from MP Biomedicals (Solon, OH, USA). The Fast green dye used to dye protein was from Allied Chemical (Cleveland, NY, USA), Nile red (99% pure) used to stain fat was from Acros Organics, and Rhodamine B used to stain polysaccharides was from Sigma Aldrich (Burlington, MA, USA). Whole milk and organic half-and-half (no added stabilizers) were used to make yogurt and sour cream, respectively. Whole milk powder was purchased from Hoosier Hill Farm (Fort Wayne, IN, USA) for use in making yogurt. Choozit^®^ buttermilk/sour cream culture containing *Lactococcus lactis* subsp. *Lactis*, *Lactococcus lactis* subsp. *cremoris*, *Lactococcus lactis* subsp. *lactis biovar. diacetylactis*, and *Leuconostoc mesenteroides* subsp. *cremoris* from Danisco (Beloit, WI, USA) was used to make the sour cream. YO-CULT culture containing *Streptococcus thermophilus* and *Lactobacillus delbrueckii* subsp. *bulgaricus* from Biena (Saint-Hyacinthe, QC, Canada) was used to make the yogurt.

### 2.2. Complex Preparation

WPI and SPI hydrolysates created by Alcalase hydrolysis: A 5% protein dispersion was made and hydrated overnight with constant stirring. The pH was then adjusted to 8.0 with 1 M NaOH and equilibrated at 55 °C. Alcalase was added at 0.044 Au/g protein and the WPI and SPI were hydrolyzed for 10 min. For the SPI and lecithin complex, the SPI was only hydrolyzed for 2 min. The hydrolysate samples were then placed in a boiling water bath for 10 min to denature the enzyme and any remaining unhydrolyzed protein. The samples were then cooled to room temperature and centrifuged at 10,000× *g* for 10 min and the supernatant was collected and lyophilized according to the standard operating procedure used in our laboratories.

High-performance liquid chromatography by size-exclusion principle (HPLC-SEC) was performed according to the method we previously reported [12] to determine the average molecular weight of the WPI and SPI hydrolysates in duplicates. The average molecular weight and relative quantity were calculated by finding the percent area of each peak and its corresponding molecular weight was compared with the standard curve.

Polysaccharide hydrolysates created by cellulase hydrolysis: Polysaccharide (LBG and LC) dispersions were made at 1% (*w*/*v*) and hydrated overnight with constant stirring. The pH was adjusted to 6.0 with 1 M HCl and equilibrated at 50 °C. Cellulase was added at 10% weight of the polysaccharides and hydrolyzed for 48 h with constant shaking. The samples were then placed in a boiling water bath for 10 min to denature the enzyme. The hydrolysates were lyophilized individually for use as a control or complexed with protein before lyophilization.

The Somogyi–Nelson method with slight modification was used to determine the reducing sugar content in the LBG and LC hydrolysates [25], as reported by Sevilla et al. [26]. A standard curve was created using the glucose standard. From the resulting linear regression equation, the content of reducing sugars was calculated for each sample. The degree of hydrolysis (DH) was calculated using the following equation:

$$DH\left(\%\right)=\left(\frac{Final\;reducing\;sugar\left(\frac{mg}{mL}\right)-Initial\;reducing\;sugar\left(\frac{mg}{mL}\right)}{Initial\;concentration\;of\;polysaccharide\left(\frac{mg}{mL}\right)}\right)\times 100$$

Protein and polysaccharide complexation through pH cycling: The pH cycling method reported by Li and Zhong [27] was used to complex the proteins and polysaccharides with slight modifications. Protein dispersions were made at 10% (*w*/*v*) and polysaccharide dispersions were made at 1% (*w*/*v*) and hydrated overnight. The protein dispersion’s pH was adjusted to 11.3 with 1 M NaOH and then mixed with the polysaccharide dispersion at a 5:1 ratio as dry matter. The pH was adjusted again to 11.3, if necessary. This mixture was then stirred for 1 h, the pH was adjusted to 7.0 with 1 M citric acid, and then heated at 50 °C in a water bath with constant stirring for 30 min. The pH was then adjusted to a final pH of 4.5 with 1 M citric acid and stirred for another hour. These samples were then lyophilized. The hydrolysate complexes were made using the same method as the unhydrolyzed complexes. There was no removal of the un-complexed protein and polysaccharides.

Formation of SPI hydrolysate and lecithin complex: SPI hydrolysates that were hydrolyzed for 2 min by Alcalase were used to prepare a complex with soy lecithin as described in Fomich et al. [24]. In brief, 5% (*w*/*v*) dispersions of SPI hydrolysates and lecithin were made and hydrated overnight. Then, calculated volumes with known concentration were mixed in a 50 mL tube to create a complex of 95% hydrolysate and 5% lecithin. To induce complexation the tubes were placed in a boiling water bath and vortexed every 2 min for 10 min. The complexes were then lyophilized before incorporation into the dairy system.

### 2.3. Complex Characterization

Determination of complex’s IRI activity using a Splat Assay: A standard Splat Assay procedure was used to quantify ice recrystallization activity of the unhydrolyzed and hydrolyzed protein–polysaccharide complexes [28]. In brief, solutions were made of 2% (*w*/*v*) of the complexes in 20 mM NaCl solution at a pH of 7.0 and 4.5. One drop of the sample solution was dropped from 1.5 m onto a pre-cooled −80 °C slide and annealed at −8 °C using a cryo-stage HCS 302 (Instec Instruments, Boulder, CO, USA) for 30 min. Three pictures of each drop were taken using polarized light microscopy (Leica, DM2700 M, Wetzlar, Germany) with a built-in digital camera (Leica, DMC 4500, Wetzlar, Germany). This served as one replicate and the measurement was performed in duplicate. The PEG of the same concentration and pH was used as a negative control. The Feret diameter was determined by using a combination of Cellpose and Fiji [29]. The average Feret diameter was calculated from two treatment replicates with each replicate having three images and measurements.

Analysis of particle size of complexes using the Zetasizer: To validate complexation of the proteins and polysaccharide, particle size analysis was performed. Selected complexes were chosen based on their IRI activity and their performance in the dairy system in the preliminary trials. Particle size distribution measurements were performed in triplicate using Malvern Zetasizer, model 3000, (Malvern Instruments, Worcestershire, UK). Samples were prepared at 1 mg/mL in 20 mM NaCl at both pH 7.0 and 4.5 and placed in a cuvette. One replicate was scanned three times. Particle size distribution was reported as a percentage of the total volume of particles in a particular size range.

Confocal microscopic observation for complexation validation: Fast green and Rhodamine B stain solutions were prepared at 1 mg/mL in DI water and Nile red was prepared at the same concentration in methanol to stain the protein, polysaccharides, and lipids, respectively. Complexes were made at 5% (*w*/*v*) in DI water with a total volume of 1 mL. Then, 20 μL of Fast green and Rhodamine B stain were added to the sample dispersion, vortexed, and incubated overnight at 4 °C. A drop of the sample was placed on a microscope slide and a cover slip was placed on top. The samples were visualized under an inverted confocal laser scanning microscope (CLSM) (Leica Microsystems, Baden-Wurttemberg, Germany) at 63× magnification with oil immersion. The Fast green was excited at 633 nm and the emission was collected between 660 and 750 nm. The Rhodamine B dye was excited at 540 nm and the emission was collected between 553 and 623 nm. For staining lipids of lecithin and dairy products, the Nile red dye was used and excited at 488 nm and the emission was collected between 520 and 590 nm.

Amphiphilicity of complexes qualitatively evaluated by emulsion stability testing: Oil in water emulsions were created with a composition of 94% DI water, 5% soybean oil, and 1% (*w*/*v*) of the complexes or controls. Nile red dye was added to the soybean oil for better visualization during testing. Tween 80 (1%) was used as a positive control, and the proteins and polysaccharides were also evaluated individually as controls. The emulsions were homogenized using a Fisherbrand™ 850 homogenizer (Fisher Scientific (Mississauga, ON, Canada) at 10,000 rpm for 3 min. Images of the emulsions were taken at 0, 10, and 30 min and phase separation was evaluated qualitatively.

### 2.4. Evaluation of Antifreeze Activity of Complexes in Dairy Products

Sour cream sample preparation and freezing conditions: Half-and-half cream (1200 mL) was heated to 30 °C while stirring and then cooled to 25 °C before 0.83 g of culture was added. The mixture was then fermented at 25 °C for ~16 h. The final pH was ~4.35. To create a smooth finished product the sour cream was mixed in a Kitchen Aid mixer on the lowest stir setting for 5 min. Sour cream was weighed out in 120 g and placed in plastic screw top containers. The lyophilized complexes were then added at 3% weight (as-is), which is equivalent to ~18% dry-weight basis. The complexes were mixed by hand until fully incorporated. The lyophilized hydrolyzed WPI and LBG complex resulted in a hard mass after complexation that needed to be rehydrated before incorporation into the sour cream. The hydrolyzed WPI and LBG complex was hydrated in 3 mL of DI water overnight before being mixed into the sour cream. The protein and polysaccharides were added individually as solids at their respective ratio (5:1) as the controls and mixed by hand until fully incorporated. A “Fresh/Frozen + Water” treatment was added as a control to account for the additional water added for hydrating the hydrolyzed WPI and LBG complex. All samples were hydrated overnight at 4 °C before being frozen at −22 °C for 4 days. The samples were then thawed overnight at 4 °C and equilibrated at room temperature for ~2–3 h before testing. All treatments were performed in duplicate.

Yogurt preparation and freezing conditions: To increase the total solids in the milk, whole milk powder was added at 3.33% (*w*/*v*) to the whole milk (1200 mL) and hydrated overnight while stirring. The milk was heated to 90 °C while constantly stirring and held at this temperature for 10 min. The milk was then cooled to ~43 °C before 0.21 g of culture was added. The mixture was fermented at 49 °C for 1 h, then the temperature was reduced to 30 °C for another 4 h, and lastly the temperature was raised to 35 °C for the last two hours to set the yogurt. The final pH was 4.6. The yogurt was then mixed in a Kitchen Aid mixer on the lowest stir setting for 1 min to produce a smooth finished product. Yogurt was weighed out in 120 g and placed in plastic screw top containers. The lyophilized complexes were then added at 3% weight (as-is), which is ~19.5% dry-weight basis, and mixed until fully incorporated. The same hydration procedure for the hydrolyzed WPI and LBG complex as used for the sour cream was used for the yogurt. The protein and polysaccharides were added individually as solids at their respective ratio (5:1) as the controls and mixed by hand until fully incorporated. All samples were hydrated overnight at −4 °C before being frozen at −40 °C for 2 days and then at −22 °C for 2 days. The samples were then thawed overnight at 4 °C and equilibrated at room temperature for ~2–3 h before testing. All treatments were performed in duplicate.

Texture analysis to evaluate freeze-induced damage: A T.A.TXT2 texture analyzer (Texture Technologies Corp., South Hamilton, MA, USA) was used to perform a single penetration test with a 2.5 cm diameter Perspex cylindrical probe and this was repeated in triplicate on a single sample. The test speed was 1.5 mm/s, the distance of penetration was 15 mm, and the trigger force was 5 g. The average firmness, cohesiveness, and consistency were recorded as the peak positive force, peak negative force, and positive area under the curve, respectively.

Lacunarity image analysis to quantify graininess due to freeze-induced damage: This method was developed to quantify the visual graininess that has been observed in sour cream and yogurt after freezing. In brief, 0.3 g of each sample was gently mixed with 1 mL of water. Different sample dilutions and light exposures were investigated to optimize the sample preparation conditions. It was determined that a 30% dilution with full light exposure produced the most consistent and accurate results. A drop was then placed under a microscope and two images were taken at 10× objective using light microscopy (Leica, DM2700 M, Wetzlar, Germany) with a built-in digital camera (Leica, DMC 4500, Wetzlar, Germany). This was repeated in duplicate for each sample. Using Fraclac of Fiji, the lacunarity of the image was measured by a sliding box method [30]. The analysis was run using the gray scale differential setting, the minimum pixel size was 5, the max size of the box was set to 45% of the image, the sliding distance was set to 5 pixels for both the X and Y direction, and a block series scaling method was used.

Qualitative analysis of freeze-induced damage by confocal laser scanning microscopy (CLSM): Fast green was used to stain protein and Nile red to stain fat in sour cream and yogurt samples to evaluate freeze-induced matrix damage. Samples of 1.0 g were weighed and diluted in 9 mL of DI water. Each dye was added at 100 μL and the samples inverted by hand to mix. The samples were left to stain overnight before being visualized under the confocal microscope. A single drop was placed on a microscope slide and a cover slip was placed on top. The samples were visualized under an inverted CLSM (Leica Microsystems, Baden-Wurttemberg, Germany) at 10× objective. The Nile red dye was excited at 488 nm and the emission was collected between 520 and 590 nm while the Fast green was excited at 633 nm and the emission was collected between 660 and 750 nm.

Syneresis measured by water separation: To evaluate syneresis of the samples, 15 mL centrifuge tubes were filled with 10 mL of yogurt or sour cream after freezing. The samples were then left to naturally settle overnight and the water that had separated from the sample was calculated as % of total volume. This was performed in duplicate.

### 2.5. Statistical Analysis

All treatments were prepared and tested in duplicate (i.e., two parallel samples from a single complexing production batch); thus, results are considered proof-of-concept and not generalizable without further complexing treatment replication. Two different fermentation batches of each of the sour cream and yogurt were used to incorporate the treatments. Results were reported as mean ± standard deviation. A nested design was used since not all of the treatments appeared in both the unhydrolyzed and hydrolyzed groups. The texture analysis data was log transformed to meet the assumption of an ANOVA that the data are normally distributed. An ANOVA test was conducted using JMP v17.0 (SAS Institute, Cary, NC, USA). Statistical significance was determined as *p* < 0.05. A Tukey HSD post hoc test was used to compare the treatment means.

## 3. Results and Discussion

### 3.1. Degree of Hydrolysis (DH) of the Polysaccharide and Protein Hydrolysates

In order to create complexes that have the potential to have increased mobility in the food matrix, the proteins and polysaccharides were tested at lower molecular weight (MW). The LBG and LC were hydrolyzed by cellulase for 48 h and DH results are shown in Table 1. The DH of both polysaccharides was low, however the reducing sugar content increased by 30 and 22 times for LBG and LC, respectively. This is an indication of sufficient breakdown of the polysaccharide. The lower-than-expected DH is most likely due to the relatively less abundant β-1,4 glycosidic bonds compared to cellulose, because cellulase is an enzyme that is specific to β-1,4-glycosidic bonds [31]. Due to this specificity, it is typically used to hydrolyze cellulose which is made of glucose monomers [31,32]. LBG is a galactomannan that has a β-1,4-mannose backbone and D-galactopyranosyl side branches linked by α-1,6- linkages [33]. Carrageenans are sulfated galactans that are made of alternating α-1,3 and β-1,4 glycosidic bonds between D-galactopyranosyl units [34].

WPI and SPI were hydrolyzed by Alcalase which is a non-specific protease. Due to the random nature of this enzyme, various MW distributions of peptides can be formed [35]. The results of the hydrolysis treatment are shown in Table 2, which shows high efficiency of hydrolysis. Multiple hydrolysis times were tested for each protein and since there were similar MW distributions among times, a time of 10 min was chosen for use in this work. WPI had more than 60% mass with less than 1 kDa and 20% between 1 and 5 kDa. The small size of these hydrolysates could potentially result in poor complexation if the polysaccharides are too large compared to the protein hydrolysates. For future work, the small peptides should be removed before making the complexes.

### 3.2. Ice Recrystallization Inhibition (IRI) Activity of Protein–Polysaccharide and Hydrolysate Complexes

The IRI activity of the protein–polysaccharide and hydrolysate complexes are shown in Table 3. Not all complexes that were tested during preliminary testing were chosen for final evaluation. This is because during preliminary testing, some samples were found not to be IRI-active in the model system but performed better in the dairy system as supported by texture analysis and vice versa. Therefore, only selected complexes were chosen for final evaluation based on the preliminary results. The average percent decrease in ice crystal size relative to PEG (negative control at the corresponding conditions) is reported in Table 3. Complex treatment and hydrolysis treatment showed a significant effect (*p* < 0.05). At both pH, the unhydrolyzed complexes of SPI and LBG as well as WPI and LBG, and the hydrolyzed SPI and lecithin complexes had the highest IRI activity as indicated by the highest reduction in ice crystal size relative to PEG. The unhydrolyzed WPI and LC complex did not show IRI activity at either pH, this was most likely due to its limited dispersibility. Except for the unhydrolyzed WPI and LC and the hydrolyzed WPI and LBG complexes, all the complexes had IRI activity even at pH 4.5 which was important to establish because this is similar to the acidic environment of the sour cream and yogurt. All the controls also proved to be inactive, indicating a synergistic effect of the proteins and polysaccharides when complexed together. However, the hydrolysate complexes did not seem to have increased IRI activity compared to the unhydrolyzed complexes as hypothesized. This result–hypothesis disagreement may indicate that when a system is well and uniformly mixed, water-binding is a more important factor than molecules’ mobility to act on the water–ice interface. From the data of the model system, all complexes should have IRI activity under the pH conditions of sour cream and yogurt to justify their evaluation in these dairy systems.

In a study reported by Gaukel et al. [20], a synergistic effect between fish antifreeze proteins and sodium alginate in a sucrose solution was seen. IRI activity was significantly increased compared to when the antifreeze protein was used alone. This synergistic effect of reducing ice recrystallization was also seen between a winter wheat grass ice structuring protein and LBG [36]. Such effect could be due to the hydration properties of the gums [20]. It was believed that the sodium alginate allowed increased interaction with water, therefore less water interacted with the antifreeze protein. This allowed for better interaction of the protein at the ice crystal interface, preventing ice crystal growth. The same theory of IRI mechanism was described in the study reported by Monalisa et al. [19] where there was a synergistic effect between AFP and corn starch. The starch was thought to have increased interaction with the water molecules, limiting the interactions between the water molecules and the AFP. This allows for better interaction of the AFP with the ice crystal surface [19]. This could be a reason for the improved IRI activity seen in our protein–polysaccharide complexes. Another possible mechanism of the complexes’ IRI activity is through the disruption of the quasi-liquid layer. If the complexes sit in this quasi-liquid layer, they can prevent ice recrystallization through accretion by inhibiting the ordering of water into an organized ice structure and merging the ice crystals [37].

### 3.3. Amphiphilicity of Complexes Determined by Emulsification and Stability

**Figure 1:**
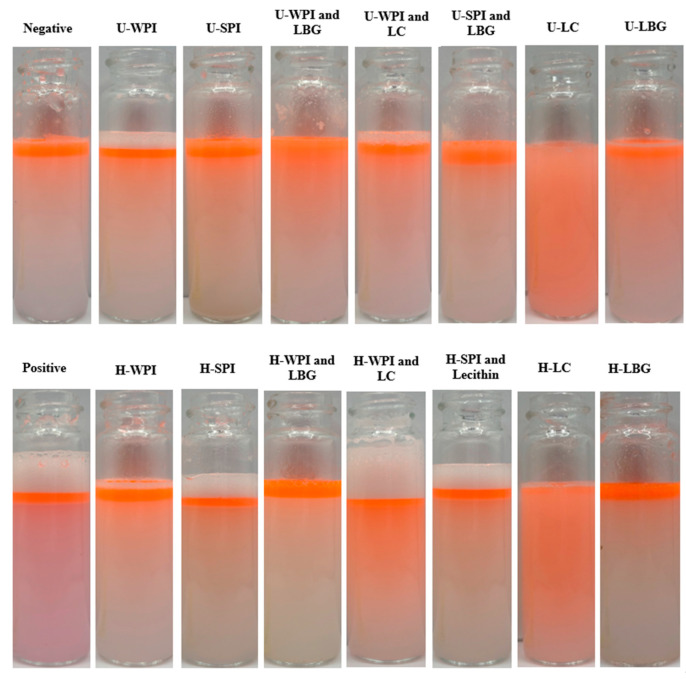
Oil in water emulsions (94% water, 5% oil, 1% (*w*/*v*) complexes) at pH 7.0 after 10 min prepared by homogenizing at 10,000 rpm for 3 min. “U-” indicates unhydrolyzed and “H-” is hydrolyzed. Negative control is only water and oil, and the positive control is oil and water with Tween 80 (1%). All samples visualized in 15 mL vials.

To explain complexes’ IRI activity, their amphiphilicity was investigated as this has been known to be a key characteristic for an enhanced IRI activity. Oil in water emulsions (94% water, 5% oil) with 1% (*w*/*v*) of the complexes, and individual proteins, polysaccharides, and hydrolysates at their proportional quantity as controls, were made to qualitatively evaluate the complexes amphiphilicity. Images of the emulsions after 10 min of settling are shown in Figure 1. The stability of emulsions was also evaluated after 30 min, but no noticeable difference was seen compared to the 10 min. The unhydrolyzed WPI and LBG and hydrolyzed WPI and LC complexes seemed to be the only ones that had relatively high emulsion stability after 10 min. The unhydrolyzed and hydrolyzed LC controls also showed very good emulsion stability after 10 min. All of the other samples had a clear separation of oil and water. The unhydrolyzed WPI and LBG complex shows better stabilization than the unhydrolyzed WPI and LBG by themselves indicating a synergistic effect. The emulsion stability for the unhydrolyzed WPI and LBG complex could indicate increased amphiphilicity of the complex. Polysaccharides have been shown to coat other macromolecules, such as plant proteins, to alter their overall hydrophobicity and colloidal stability [38]. The hydrolyzed WPI and LC complex also showed increased foaming compared to other samples. In a study performed by Jarpa-Parra et al. [39], foam stability was evaluated using lentil legumin protein and various polysaccharides. At pH 5, an increased foam stability was observed with the protein–polysaccharide complexes compared to the legumin alone [39]. It was suggested that the protein and polysaccharide aggregates increased the viscosity of the interfacial layer and created a thick interface that exhibited low gas permeability [39]. This led to reduced foam drainage and therefore increased foam stability. This could be the support for the hydrolyzed WPI and LC complex having increased foam capacity and stability. This could also be due to the LC as its stability was equivalent to the complex.

Since amphiphilicity has been found to be an important factor for IRI activity, this improved amphiphilicity indicated by emulsion stability could be an explanation behind the observed IRI activity of the unhydrolyzed WPI and LBG complexes. However, these data do not strongly support the finding for all the complexes, since some of the complexes that had high IRI activity showed poor emulsion stability, such as the unhydrolyzed SPI and LBG and the hydrolyzed SPI and lecithin complexes as shown in Figure 1. Therefore, amphiphilicity may just be one of the many factors that contribute to IRI activity. The relative importance of molecular characteristics and their water-binding ability can be a debatable topic. In addition, the environmental factors (media conditions) can impact these properties significantly.

### 3.4. Particle Size Analysis of Unhydrolyzed and Hydrolyzed Complexes

**Figure 2:**
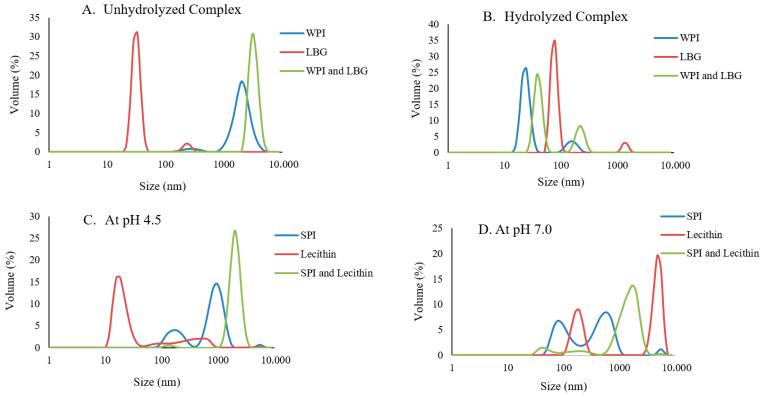
Particle size distribution of unhydrolyzed WPI and LBG complex and controls (**A**) and hydrolyzed WPI and LBG complex and controls (**B**) at pH 7 in 20 mM NaCl, and hydrolyzed SPI and lecithin complex and controls at pH 4.5 (**C**) and pH 7.0 (**D**) in 20 mM NaCl. Confocal image evaluation for complexation validation.

To validate complexation of the protein and polysaccharide complexes, their particle size and distribution were evaluated. Only selected complexes including the hydrolyzed SPI and lecithin complex, the hydrolyzed WPI and LC complex, and the unhydrolyzed and hydrolyzed WPI and LBG complexes were investigated because of their IRI activity and performance evaluation in the dairy system. The average particle diameter of the complexes and their individual counterparts’ sizes are shown in Table 4. At pH 4.5, all the complexes along with the unhydrolyzed WPI and LBG at pH 7.0 seemed to have a larger particle diameter than their individual counterparts which validates complexation between the protein and polysaccharides and lecithin. The particle size distribution profiles of the unhydrolyzed WPI and LBG at pH 7.0 and the SPI and lecithin complex at 4.5 are shown in Figure 2A,C, respectively, to demonstrate this observation. The hydrolyzed protein, such as WPI, can aggregate at pH 4.5 due to the exposure of hydrophobic areas during hydrolysis, resulting in a larger particle size [40]. The complexes at pH 4.5 seem to be larger than at pH 7. This could be due to aggregation since the pH is slightly below the isoelectric point of the proteins. The smaller particle size at pH 7.0 is most likely due to increased solubility because of the protein carrying a net negative charge at pH above its isoelectric point. At pH 7.0 there was not as clear of a particle size trend for the complexes. The unhydrolyzed WPI and LBG complex was bigger than their individual counterparts indicating complexation. The SPI and lecithin and the hydrolyzed WPI and LBG complexes were the only complexes larger than their protein counterpart. The size distribution profiles of the hydrolyzed WPI and LBG complexes and the SPI and lecithin at pH 7 are shown in Figure 2B,D, respectively. The majority of the complexes, except the unhydrolyzed WPI and LBG complex, show bimodal curves in between the particle size range of the individual biopolymers. This could result in an average particle size that is smaller than the individual biopolymers as shown in Table 4. The difference in particle size distribution could indicate that there is a difference in degree of complexing.

**Figure 3:**
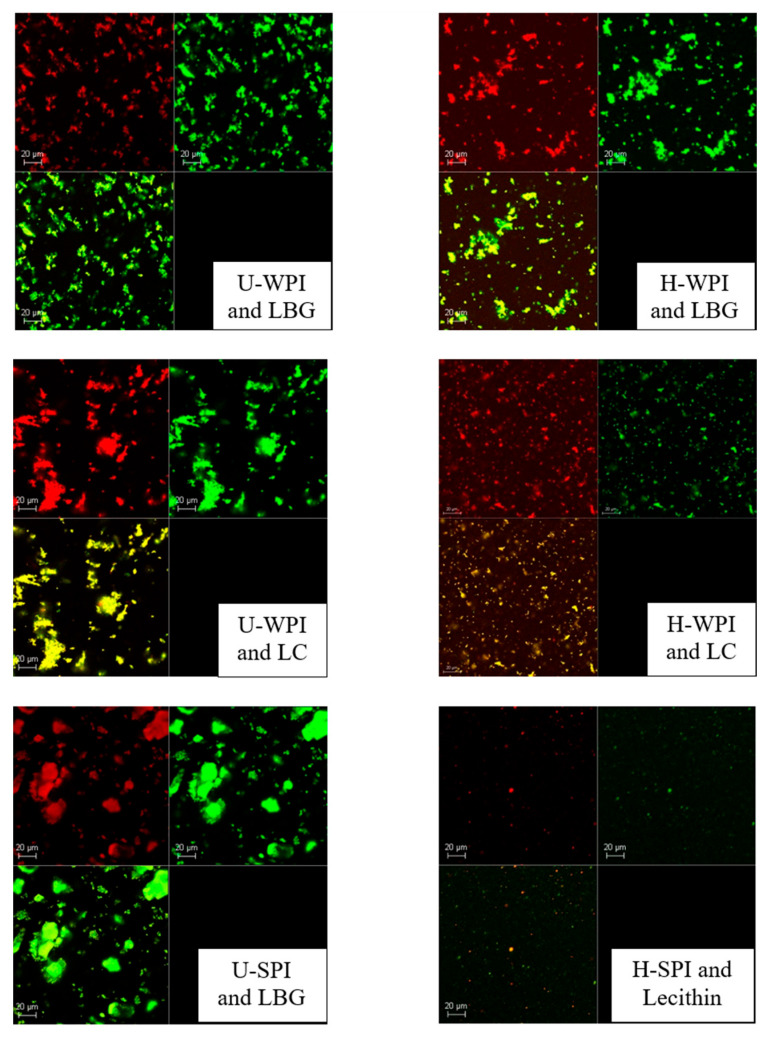
Confocal images of protein and polysaccharide complexes at 5% (*w*/*v*). Green indicates protein and red indicates polysaccharide or lecithin. In each set of pictures, the bottom left image is the top two overlayed. “U-” indicates unhydrolyzed and “H-” indicates hydrolyzed. Samples were visualized at 63× objective, and scale bars are 20 microns.

Images of the protein and polysaccharide complexes along with the SPI and lecithin complexes can be seen in Figure 3. The green represents protein stained with Fast green while the red represents polysaccharide or lecithin either stained with Rhodamine B or Nile red, respectively. The unhydrolyzed complexes showed minimal image discrepancy when the two wavelengths were overlayed indicating good complexation between the protein and polysaccharide. There were no defined or unique aggregation patterns seen either, all the unhydrolyzed complexes showed small, dispersed aggregates.

The hydrolyzed complexes showed a small amount of free polysaccharide indicating that the degree of complexation was not as good compared to the unhydrolyzed. This could be due to the small size of the WPI hydrolysates being unable to fully complex with the LBG and LC. However, overall, there still seemed to be good complexation due to the majority of the green protein overlapping with the red polysaccharide. The hydrolyzed complexes showed similar aggregation characteristics as the unhydrolyzed complexes. The SPI and lecithin complex showed different characteristics than what Fomich et al. [24] reported, in which fibril formation between the SPI hydrolysates and lecithin was observed. There was also more free lecithin seen, indicating that the SPI and lecithin might not be fully complexed or there is an excess quantity of lecithin.

The formation of similar complexes between WPI and LBG or LC were made and tested in the same research laboratory using aqueous solutions and cream cheese systems [26]. FTIR was used as a validation to confirm the complex formation. The peak shift and intensity changes in the complexes compared to the individual biopolymers suggested interactions and complexing, so FTIR was not performed for this work.

### 3.5. Effect of Protein–Polysaccharide Complexes on Freeze-Induced Damage in Sour Cream and Yogurt

Yogurt and sour cream composition: Whole milk yogurt was made from whole milk with added whole milk powder and resulted in approximately 4.25% fat, 4.25% protein, 6.84% carbohydrates, and a moisture content of 84.66%. Sour cream was made from organic half-and-half cream and resulted in roughly 10.00% fat, 3.33% protein, 3.33% carbohydrates, and a moisture content of 83.34%. These compositions were calculated from the nutrition label of the cream and milk used. Based on these moisture contents, the complexes were added at ~18% and ~19.5% dry-weight basis for the sour cream and yogurt, respectively.

Texture analysis to quantify freeze-induced damage: The firmness, cohesiveness, and consistency of the sour cream and yogurt after freezing and thawing was measured by texture analysis and reported in Table 5 and Table 6, respectively. After freezing, there was a significant decrease in firmness, cohesiveness, and consistency in all unhydrolyzed complex treatments for both the sour cream and yogurt, similar to the controls. In the sour cream the hydrolyzed complexes performed similarly to the unhydrolyzed complexes. Therefore, the treatments were not effective at preventing freeze-induced damage. These data do not support the original hypothesis that the hydrolyzed complexes would perform better due to their increased mobility in the food matrix, and the possible reason is discussed in the previous section. Overall, the difference in composition between the sour cream and yogurt did not seem to influence the effectiveness of the complexes as the textural quality was not maintained in either product.

In yogurt a slightly different trend was observed in the texture analysis with the hydrolyzed complexes. The hydrolyzed WPI and LBG complex and the SPI and lecithin complex resulted in decreased firmness, cohesiveness, and consistency in the yogurt. These results are similar to those of unhydrolyzed complexes. However, the hydrolyzed WPI and LC complex retained similar textural properties compared to the fresh yogurt. After freezing, the hydrolyzed WPI and LC complex had similar firmness, cohesiveness, and consistency compared to the fresh samples. This complex also performed better than the two biopolymer controls indicating a synergistic effect.

There has been little to no research reporting on the effect of freeze-induced damage on the textural qualities of yogurt and sour cream. Most research has focused on low- and high-moisture mozzarella cheese [4,8]. Such decreases in firmness and cohesiveness after freezing as observed in this study were also reported by Alinovi and Muchetti [8] and To et al. [4] in mozzarella. This was related to protein dehydration and leakage of fat from the matrix which can leave large serum channels resulting in a more porous structure [4,41,42].

**Figure 4:**
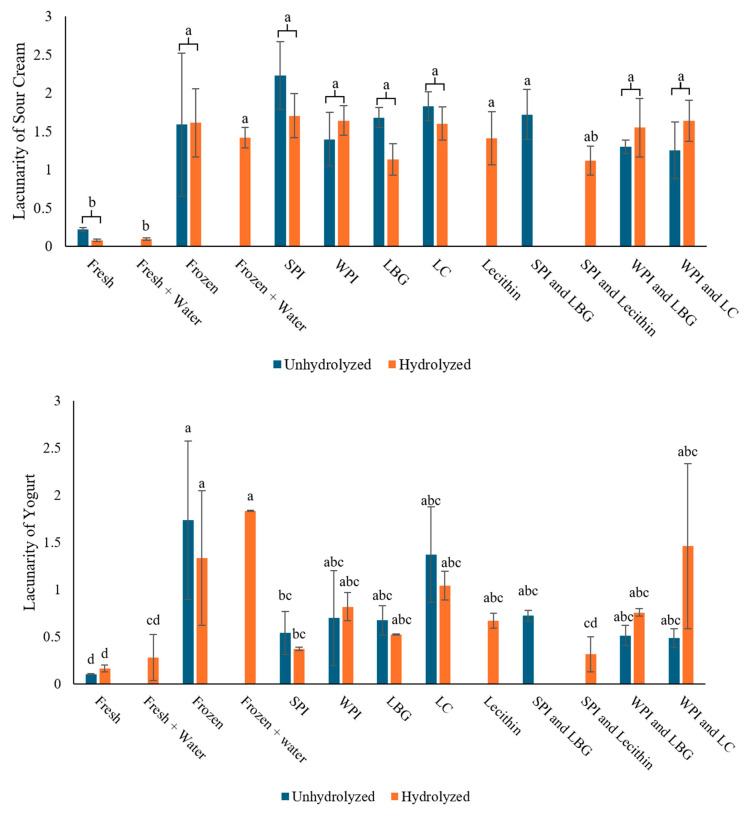
Lacunarity of frozen thawed sour cream (**top**) and yogurt (**bottom**) with unhydrolyzed and hydrolyzed complexes and controls. Standard deviation was from two treatment replicates each having three images evaluated. Mean comparison letters for the sour cream only show the main effect of treatment since the interaction of hydrolysis and treatment was not significant. Different letters indicate significantly different treatments (*p* < 0.05).

Lacunarity of freeze-induced damage: After freezing, the sour cream and yogurt were much thinner and grainy. The graininess observed in the sour cream and yogurt with unhydrolyzed and hydrolyzed complexes after freezing was quantified using microscopic image analysis and the lacunarity values are reported in Figure 4. Lacunarity is a term used to describe the distribution of spaces and gaps [43]. An image that is homogeneous in gaps or has fewer spaces in the pattern is described as having low lacunarity. Whereas, if an image has irregular or heterogeneous gaps and spacing, it has high lacunarity. There was significant difference among treatments, but no significance difference between unhydrolyzed and hydrolyzed complex treatments. In the sour cream samples, both the unhydrolyzed and hydrolyzed complexes, as well as all the controls, resulted in increased lacunarity after freezing. There were large particles after freezing, assumed to be due to protein aggregation, and the sample was less homogeneous. This graininess, as mentioned in the texture analysis results, is caused by conformational changes in the proteins during freezing.

A similar trend was seen in yogurt. The samples with the unhydrolyzed and hydrolyzed complexes, along with all the controls, had increased lacunarity after freezing compared to the fresh samples. This is an indication of freeze-induced damage. However, the complex treatments had reduced degree of lacunarity compared to the frozen control (with nothing added) indicating that our treatments did help reduce the graininess caused by freezing. The samples with the hydrolyzed complexes behaved similarly to the unhydrolyzed complexes. However, the SPI and lecithin and WPI and LBG complexes had decreased lacunarity compared to the frozen control samples. Despite the hydrolyzed WPI and LC having retained similar textural properties as the fresh yogurt as discussed previously; it still had a high level of graininess which would make it unacceptable for USDA and customer standards.

The sour cream seemed to have higher lacunarity or increased graininess compared to the yogurt. The difference in effectiveness of the complexes in sour cream and yogurt could be due to its different composition. Sour cream has less protein, which is the main structuring agent in milk gels, than yogurt. With less protein and with complexes that are not highly effective at reducing freeze-induced damage, this can leave the sour cream without a sufficient structure to maintain after freezing. In yogurt, the increased protein content leaves more structure that can be enhanced by the complexes during freezing.

**Figure 5:**
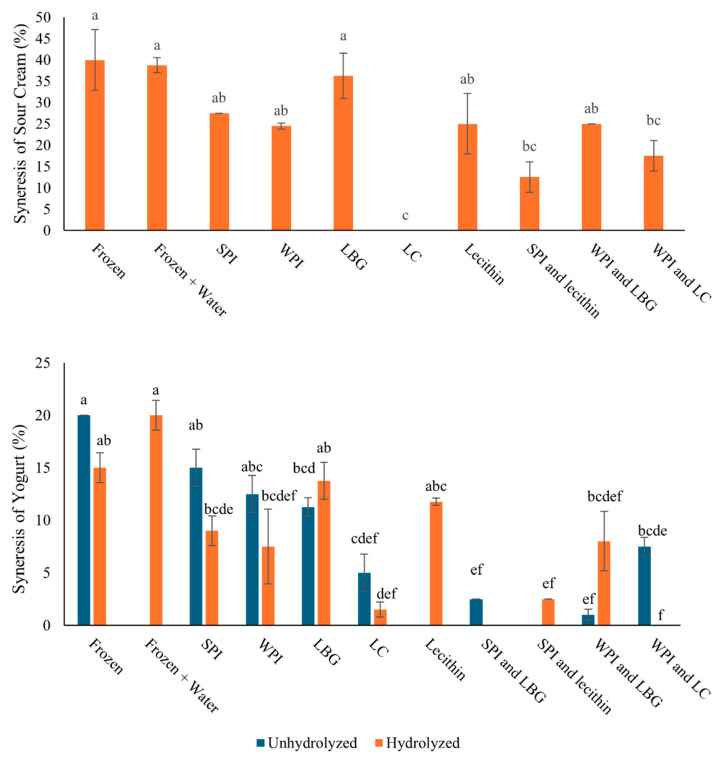
Syneresis, expressed as water (%) relative to the total volume of sour cream (**top**) frozen with hydrolyzed complexes and yogurt (**bottom**) frozen with unhydrolyzed and hydrolyzed complexes. Standard deviation was between two replicates. Different letters indicate significantly different treatments (*p* < 0.05).

Degree of syneresis: Syneresis can occur when protein becomes denatured or damaged and is no longer able to hold water as effectively. After freezing, there was increased syneresis, as mentioned previously. Figure 5 shows the amount of water leaked from sour cream with hydrolyzed complexes and yogurt with unhydrolyzed and hydrolyzed complexes along with controls. The unhydrolyzed treatments of the sour cream were not shown due to their disposal before this method was established. For all the complexes in both systems there was a decrease in syneresis compared to the control which had nothing added to it. Specifically, in the yogurt sample with the hydrolyzed WPI and LC complex there was no water separation observed. This is most likely due to hydrolyzed polysaccharide having an increased water holding capacity as seen in the LC control. There was also a synergistic effect when it was complexed with the hydrolyzed WPI. This synergistic effect was also seen between the hydrolyzed SPI and lecithin complex.

The graininess and syneresis of sour cream and yogurt after freezing could be due to protein aggregation as the result of conformational changes in the proteins during freezing. During freezing, proteins can undergo conformational changes that would expose hydrophobic groups. These newly exposed hydrophobic patches could result in aggregation of the proteins by hydrophobic interaction and contribute to the graininess observed after freezing. The increased syneresis could also be due to the decrease in the protein’s water holding capacity [3,5]. After suffering freeze-induced damage, proteins are unable to rebind water resulting in increased unbound water or syneresis.

**Figure 6:**
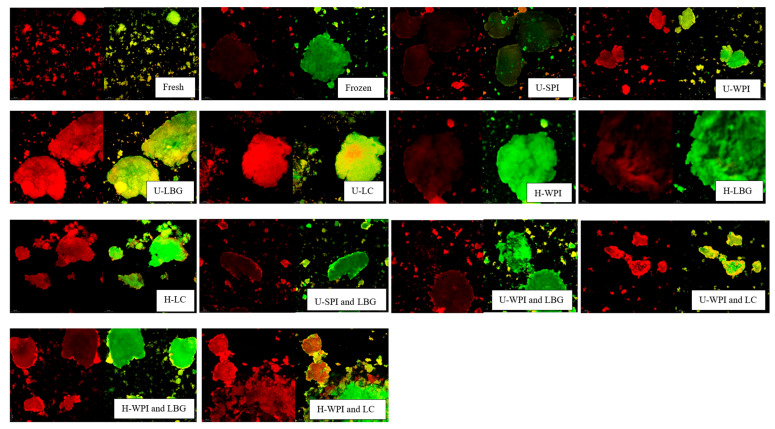
Confocal images of fresh and frozen yogurt with unhydrolyzed (U) and hydrolyzed (H) complexes and controls added at 3% as-is weight basis. Samples visualized at 10× objective. Red indicates fat and green indicates protein. This scale bar size 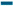 represents 20 microns.

Qualitative microstructure analysis of freezing-induced damage: The microstructure of yogurt before and after freezing with unhydrolyzed and hydrolyzed complexes are shown in Figure 6. The green areas indicate protein, and the red indicates fat. In the fresh samples, the fat is enclosed by the protein which shows small particles that are evenly distributed. After freezing, all treatments had larger protein particles, which supports the concept that the graininess seen in the frozen yogurt is due to protein aggregation. Interestingly, even after freezing there was minimal fat leakage from the protein except for the WPI and LC complex treatment, which suggests that the milk fat globules were not severely ruptured due to ice recrystallization. This indicates that the main freeze-induced damage is protein aggregation. The hydrolyzed complexes showed a similar result as the unhydrolyzed. After freezing, there was a large amount of protein aggregation resulting in fewer and larger aggregates in the field of view.

**Figure 7:**
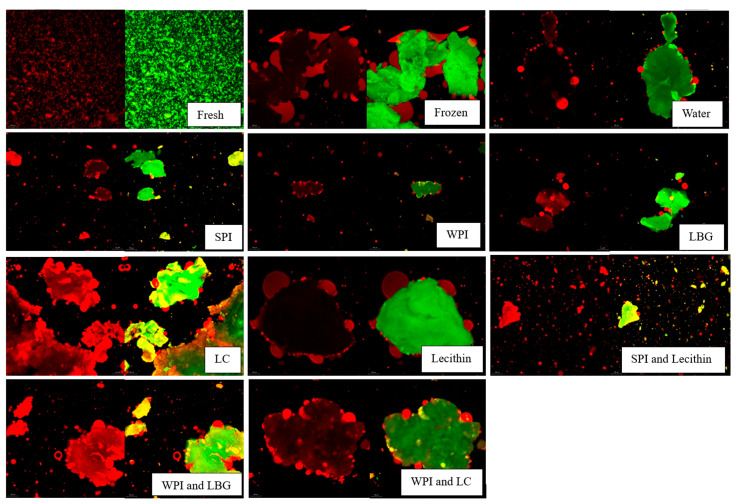
Confocal images of fresh and frozen sour cream with hydrolyzed complexes added at 3% weight as-is. Samples visualized under 10× objective. Red indicates fat and green indicates protein. This scale bar size 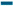 represents 20 microns.

The microstructure of the sour cream after freezing showed slightly different results than the yogurt. Figure 7 shows sour cream before and after freezing with hydrolysate complexes. The effect of unhydrolyzed complexes on sour cream’s microstructure after freezing was not evaluated due to the disposal of samples before this technique was developed. The fresh sour cream showed very small protein aggregates that were homogeneously distributed, and the fat was completely enclosed within the protein matrix. The frozen samples showed large irregular distributed protein aggregates. However, different from yogurt, fat leakage from the protein matrix can be seen. Sour cream contains more fat that may have contributed to the higher degree of fat droplet damage. The rupture of the milk fat globular membrane and protein matrix is likely to be caused by ice recrystallization and the aggregation of proteins on the outer surface of the fat droplets [5].

Overall, this work presents a significant discrepancy for the anti-freezing efficacy evaluation using the conventional model system versus real foods. The ineffective results obtained by using foods are likely due to the complex interaction among different food components that may have interfered with the antifreeze action by the tested compounds. Abundant dairy components, such as calcium ion, lactose, phosphate, and native dairy proteins and peptides, may interact with the added complex; pH variation may alter complex structure and interactions; and cryo-concentration may exclude complexes associated with other components from interfacial regions that are important for ice growth. Therefore, future work should be conducted in simplified models and tests carried out one factor at a time to further understand how IRI agents act in a real food system. Also, for future application work, such protein–polysaccharide complexes that have demonstrated anti-freezing effectiveness in model systems should be evaluated using relatively low-moisture dairy products that may lead to improved effectiveness than for the two high-moisture products used for this study.

There are a few limitations for this work. First, all treatments applied to the two dairy products were only conducted two times, with each sample or treatment having triplicate measurements. Therefore, the statistical analysis and conclusion are relatively weak. We consider this work as pilot or a proof-of-concept report. Some of the promising treatments should be repeated with many more replicates, particularly for a heterogenous system such as dairy products. Another significant limitation is that the complexes were added at 3% as-is basis that corresponds to 18–19.5% on a dry matter basis of the two high-moisture dairy products. This greatly exceeds the typical functional additive level, and at such high concentration, textural and functional changes may reflect bulking or phase behavior changes rather than specific IRI activity. For future work, a lower addition level, and dose–response (e.g., 0.1, 0.5, 1.0, 3.0%) effect should be examined.

## 4. Conclusions

The unhydrolyzed and hydrolyzed protein and polysaccharide complexes demonstrated IRI activity in the model system, but when applied in high-moisture dairy products as a proof-of-concept test, they failed to prevent freeze-induced damage when used at high dosage (3% as-is). The sour cream and yogurt both became less firm, cohesive, and had decreased consistency under all treatments, except for the hydrolyzed WPI and LC complex. While the hydrolyzed WPI and LC complex preserved macroscopic textural parameters in yogurt, it did not prevent microstructural degradation (elevated lacunarity), underscoring the need for multi-scale evaluation in cryoprotection studies. This loss of texture quality is most likely due to protein aggregation induced by ice recrystallization. This was shown by confocal microscopy where bigger protein aggregates were observed after freezing compared to the fresh controls. This study demonstrates the newfound IRI activity of WPI and SPI complexed with LBG and LC along with SPI and lecithin, but they lack effectiveness in prevention of freeze-induced damage in the dairy systems tested. Their efficacy at realistic dosages as additives remains unproven. Nonetheless, this work demonstrates an important fact that when exploring new IRI-active molecules intended for use as antifreeze additives in foods, such additives should be proven in the multi-component matrix to validate their effectiveness and illustrate the mechanism of action.

## Figures and Tables

**Table 1:** Reducing sugar content after 48 h cellulase hydrolysis of LBG and LC samples (of 10 mg/mL) and the calculated degree of hydrolysis (DH).

	Reducing Sugar Content (mg/mL)		DH (%)
	Unhydrolyzed	Hydrolyzed	
LBG	0.01 ± 0.00 ^c^	0.30 ± 0.00 ^a^	2.86 ^a^
LC	0.01 ± 0.00 ^c^	0.22 ± 0.01 ^b^	2.08 ^b^

Data presented as mean ± standard deviation. Means of DH and reducing sugar content were compared separately; different letters indicate treatments which were significantly different (*p* < 0.05).

**Table 2:** Native protein and hydrolysates’ molecular weight distribution determined by HPLC-SEC.

	Peptide Size Distribution (%)				
Protein	Time (min)	<1 kDa	1–5 kDa	5–10 kDa	>10 kDa
SPI	0	20.8	18.9	9.3	51.1
	2	59.0	25.9	9.5	5.7
	5	66.6	19.4	10.6	3.3
	10	54.6	26.1	9.1	10.2
WPI	0	0.3	19.8	2.0	78.0
	2	51.8	25.7	10.9	11.7
	5	52.4	25.8	9.9	11.9
	10	65.3	20.8	7.8	8.3

**Table 3:** IRI activity of unhydrolyzed and hydrolyzed complexes tested at 2% in 20 mM NaCl at pH 4.5 and 7.0.

	Ice Crystal Size Reduction Relative to PEG (%)			
	pH 4.5		pH 7.0	
Controls	Unhydrolyzed	Hydrolyzed	Unhydrolyzed	Hydrolyzed
SPI	−17.94 ± 9.60 ^c^	−15.70 ± 12.19 ^c^	10.50 ± 14.51 ^cd^	7.71 ± 7.91 ^cde^
WPI	−15.35 ± 0.55 ^c^	−15.72 ± 7.74 ^c^	1.43 ± 2.80 ^cde^	3.55 ± 7.72 ^cde^
LBG	−27.16 ± 16.11 ^c^	−28.89 ± 9.40 ^c^	7.24 ± 4.65 ^cde^	−26.92 ± 7.81 ^e^
LC	−30.69 ± 8.50 ^c^	−26.69 ± 4.31 ^c^	−2.10 ± 3.66 ^cde^	16.23 ± 7.05 ^cd^
Lecithin	−39.11 ± 10.71 ^c^	—	−3.25 ± 18.36 ^cde^	—
Complexes				
SPI and LBG	37.80 ± 7.69 ^a^	—	30.74 ± 13.36 ^abc^	—
WPI and LBG	33.51 ± 19.60 ^ab^	−6.45 ± 9.29 ^bc^	63.91 ± 3.28 ^a^	−1.15 ± 3.88 ^cde^
WPI and LC	−19.71 ± 6.80 ^c^	0.79 ± 9.50 ^abc^	−8.36 ± 9.01 ^cde^	21.51 ± 5.82 ^bcd^
SPI and Lecithin	—	27.43 ± 4.25 ^ab^	—	54.98 ± 2.51 ^ab^

Empty cells indicated by “—” were treatments that were not chosen to be further investigated due to their poor performance in preliminary testing. Standard deviation is between the average of three images from two replicate analyses of each treatment. Means were compared within each pH group including both unhydrolyzed and hydrolyzed treatments; different letters indicate treatments which were significantly different (*p* < 0.05).

**Table 4:** Particle size of protein and polysaccharide dispersions (1 mg/mL) at pH 4.5 and 7.0 in 20 mM NaCl.

	Mean Particle Diameter (nm)			
	pH 4.5		pH 7	
Controls	Unhydrolyzed	Hydrolyzed	Unhydrolyzed	Hydrolyzed
SPI	—	332 ± 26 ^e^	—	245 ± 6 ^f^
WPI	2113 ± 61 ^c^	2609 ± 154 ^c^	574 ± 45 ^cde^	274 ± 88 ^ef^
LBG	1053 ± 289 ^d^	1168 ± 521 ^d^	1069 ± 218 ^bcd^	3260 ± 1069 ^a^
LC	—	713 ± 180 ^d^	—	2216 ± 1331 ^ab^
Lecithin	263 ± 2 ^e^	—	1207 ± 293 ^bc^	—
Complexes				
SPI and Lecithin	—	1095 ± 130 ^d^	—	382 ± 17 ^ef^
WPI and LBG	10,174 ± 2230 ^a^	5890 ± 1614 ^ab^	4196 ± 733 ^a^	487 ± 110 ^def^
WPI and LC	—	3174 ± 78 ^bc^	—	493 ± 44 ^def^

“—” indicate treatments that were not chosen for analysis based on preliminary data. Standard deviation is between three replicates from one sample. Mean comparison was evaluated within each pH group including both unhydrolyzed and hydrolyzed treatments and different letters indicate significantly different treatments (*p* < 0.05).

**Table 5:** Texture analysis from a single penetration test of frozen thawed sour cream with unhydrolyzed and hydrolyzed protein–polysaccharide complexes added at 3% weight (as-is).

	Firmness (g)		Cohesiveness (g)		Consistency (g.sec)	
Controls	Unhydrolyzed	Hydrolyzed	Unhydrolyzed	Hydrolyzed	Unhydrolyzed	Hydrolyzed
Fresh	53.35 ± 0.42 ^a^	64.41 ± 8.70 ^a^	−32.89 ± 0.82 ^a^	−41.70 ± 4.98 ^a^	333.81 ± 0.67 ^ab^	385.33 ± 29.98 ^a^
Fresh + Water	—	60.14 ± 8.98 ^a^	—	−36.62 ± 6.44 ^a^	—	360.35 ± 45.52 ^a^
Frozen	25.92 ± 0.28 ^cd^	29.67 ± 0.23 ^bc^	−3.53 ± 0.25 ^bcd^	−3.51 ± 0.11 ^bcd^	110.30 ± 1.24 ^def^	115.43 ± 2.34 ^def^
Frozen + Water	—	22.93 ± 2.99 ^cd^	—	−3.35 ± 0.00 ^bcd^	—	108.73 ± 4.33 ^def^
SPI	15.66 ± 0.90 ^e^	16.81 ± 0.40 ^de^	−3.08 ± 0.17 ^d^	−3.49 ± 0.14 ^bcd^	99.46 ± 3.58 ^f^	99.10 ± 2.12 ^f^
WPI	15.96 ± 0.93 ^de^	17.15 ± 0.31 ^de^	−3.65 ± 0.51 ^bcd^	−3.59 ± 0.00 ^bcd^	97.46 ± 3.16 ^f^	99.42 ± 2.28 ^f^
LBG	15.54 ± 0.37 ^e^	18.83 ± 1.78 ^cde^	−3.79 ± 0.34 ^bcd^	−3.63 ± 0.17 ^bcd^	98.04 ± 1.46 ^f^	100.71 ± 2.84 ^f^
LC	31.07 ± 12.20 ^bc^	58.13 ± 5.79 ^a^	−5.51 ± 0.23 ^b^	−5.29 ± 0.48 ^bc^	148.99 ± 35.83 ^cd^	247.27 ± 28.59 ^b^
Lecithin	—	18.99 ± 1.04 ^cde^	—	−3.20 ± 0.11 ^cd^	—	103.04 ± 1.12 ^ef^
Complexes						
SPI and LBG	16.36 ± 2.29 ^de^	—	−5.59 ± 2.32 ^bc^	—	104.08 ± 9.45 ^ef^	—
SPI and Lecithin	—	15.04 ± 0.37 ^e^	—	−3.29 ± 0.20 ^bcd^	—	100.42 ± 2.59 ^f^
WPI and LBG	15.44 ± 0.71	16.04 ± 0.59 ^de^	−4.79 ± 0.73 ^bcd^	−3.49 ± 0.08 ^bcd^	98.78 ± 2.60 ^f^	97.09 ± 0.21 ^f^
WPI and LC	44.00 ± 8.56 ^ab^	29.61 ± 2.29 ^bc^	−4.77 ± 1.16 ^bcd^	−4.47 ± 0.00 ^bcd^	163.76 ± 13.50 ^c^	139.31 ± 4.88 ^cde^

Sour cream was frozen for 4 days at −20 °C. Standard deviation is from duplicate treatments, each having three measurements. The means of the unhydrolyzed and hydrolyzed samples were compared within each texture parameter; different letters indicate significantly different treatments (*p* < 0.05). “—” is for missing data due to (1) the unhydrolyzed complexes not needing an extra water control because of their good dispersibility in the dairy system and (2) some treatments not being chosen due to their poor performance in preliminary experiments and testing.

**Table 6:** Texture analysis from a single penetration test of frozen thawed yogurt with unhydrolyzed and hydrolyzed protein–polysaccharide complexes added at 3% weight (as-is).

	Firmness (g)		Cohesiveness (g)		Consistency (g.sec)	
Controls	Unhydrolyzed	Hydrolyzed	Unhydrolyzed	Hydrolyzed	Unhydrolyzed	Hydrolyzed
Fresh	28.16 ± 4.63 ^abc^	33.15 ± 3.39 ^ab^	−13.88 ± 3.53 ^a^	−15.62 ± 1.86 ^a^	170.71 ± 33.03 ^ab^	204.75 ± 22.18 ^ab^
Fresh + Water	—	28.49 ± 3.08 ^abc^	—	−13.82 ± 2.03 ^a^	—	177.07 ± 22.85 ^ab^
Frozen	16.10 ± 1.13 ^ef^	14.36 ± 0.31 ^f^	−2.96 ± 0.11 ^d^	−3.31 ± 0.23 ^d^	104.81 ± 1.36 ^d^	96.22 ± 1.32 ^d^
Frozen + Water	—	15.34 ± 0.56 ^f^	—	−2.88 ± 0.23 ^d^	—	103.19 ± 0.64 ^d^
SPI	14.88 ± 0.14 ^f^	15.06 ± 0.23 ^f^	−3.06 ± 0.31 ^d^	−3.35 ± 0.56 ^d^	103. 04 ± 0.04 ^d^	103.48 ± 0.91 ^d^
WPI	14.98 ± 0.11 ^f^	14.82 ± 0.11 ^f^	−3.08 ± 0.11 ^d^	−3.41 ± 0.54 ^d^	103.30 ± 0.08 ^d^	101.59 ± 0.33 d
LBG	14.78 ± 0.11 ^f^	15.56 ± 0.14 ^f^	−3.87 ± 0.06 ^cd^	−3.37 ± 0.03 ^d^	100.86 ± 1.00 ^d^	105.41 ± 0.59 ^d^
LC	25.76 ± 4.41 ^bcd^	21.07 ± 0.25 ^cde^	−6.03 ± 0.45 ^bc^	−4.33 ± 0.37 ^cd^	151.61 ± 23.57 ^bc^	123.26 ± 0.54 ^cd^
Lecithin	—	15.46 ± 0.40 ^f^	—	−3.00 ± 0.45 ^d^	—	104.30 ± 1.88 ^d^
Complexes						
SPI and LBG	14.94 ± 0.51 ^f^	—	−4.47 ± 0.11 ^cd^	—	100.45 ± 4.04 ^d^	—
SPI and Lecithin	—	16.12 ± 0.08 ^ef^	—	−4.35 ± 0.06 ^cd^	—	105.68 ± 2.46 ^d^
WPI and LBG	15.10 ± 0.11 ^f^	14.70 ± 0.28 ^f^	−4.55 ± 0.17 ^cd^	−3.28 ± 0.11 ^d^	101.12 ± 0.20 ^d^	100.95 ± 0.57 ^d^
WPI and LC	19.13 ± 2.20 ^def^	37.18 ± 4.35 ^a^	−4.45 ± 0.65 ^cd^	−10.28 ± 3.47 ^ab^	116.10 ± 9.59 ^cd^	218.47 ± 30.76 ^a^

Yogurt was frozen for 2 days at −40 °C and 2 days at −20 °C. Standard deviation is from duplicate treatments; each treatment had three measurements. The means of the unhydrolyzed and hydrolyzed samples were compared within each texture parameter; different letters indicate significantly different treatments (*p* < 0.05). For “—”, see Table 5 footnote.

## Data Availability

The original contributions presented in the study are included in the article, further inquiries can be directed to the corresponding author.
